# Chronic pain after Shouldice versus Lichtenstein inguinal hernia repair: a systematic review and meta-analysis

**DOI:** 10.1007/s10029-026-03702-x

**Published:** 2026-05-12

**Authors:** Lucas Bastos Silva Carvalho, Eduardo Fagundes Muricy, Matheus Mueller Habib, Lucca Szwarcwing Barros, Maria Luiza Alves de Lima

**Affiliations:** 1 Faculdade de Medicina da Bahia, Universidade Federal da Bahia (UFBA), Praça XV de Novembro, s/n - Largo do Terreiro de Jesus Salvador, Salvador, Bahia 40026-010 Brazil; 2 Escola Bahiana de Medicina e Saúde Pública, Salvador, BA Brazil

**Keywords:** Inguinal Hernia, Chronic pain, Shouldice repair, Lichtenstein repair, Herniorrhaphy, Meta-Analysis

## Abstract

**Purpose:**

The Lichtenstein (mesh) and Shouldice (non-mesh) techniques are the main options for open inguinal hernia repair. This systematic review and meta-analysis compared the prevalence of chronic postoperative pain between them.

**Methods:**

A systematic search was conducted in Medline, Embase and Cochrane databases for studies comparing the prevalence of chronic pain after Shouldice and Lichtenstein techniques in adult patients undergoing inguinal hernia repair. Meta-analysis was performed using RevMan and the effect model was determined based on heterogeneity, assessed by the I² statistic. Subgroup analyses were conducted by mean follow-up duration and study design.

**Results:**

Ten studies with 4,122 patients (59.8% Lichtenstein) were included. Shouldice repair was associated with a 34% relative risk reduction in chronic postoperative pain compared to Lichtenstein (RR 0.66; 95%CI 0.56–0.78; *p* < 0.00001; I² = 5%). Conversely, the Shouldice technique demonstrated a higher risk of long-term hernia recurrence (RR 2.54; 95%CI 1.15–5.63; *p* = 0.02). There were no statistically significant differences between the two techniques regarding early postoperative complications (RR 1.01; 95%CI 0.85–1.19; *p* = 0.95) or mean operative time (MD 5.96 min; 95%CI -0.79 to 12.71; *p* = 0.08).

**Conclusion:**

Although the Lichtenstein repair is more widely practiced, the Shouldice technique yields significantly lower rates of chronic postoperative pain, albeit with a higher risk of long-term recurrence. This trade-off highlights Shouldice as a potential alternative in selected patients.

**Supplementary Information:**

The online version contains supplementary material available at 10.1007/s10029-026-03702-x.

## Introduction

Inguinal hernia repair is one of the most common surgical procedures performed worldwide, with millions of cases annually [1, 2]. The choice of surgical technique has important implications not only for hernia recurrence but also for postoperative morbidity, especially chronic pain, which affects long-term patient quality of life [3–5]. Among the open surgical techniques, the Lichtenstein tension-free mesh repair and the Shouldice non-mesh tissue-based repair remain the most widely performed and studied approaches [1, 2, 6, 7].

The Shouldice technique, developed in the mid-20th century, involves a meticulous multilayered reconstruction of the posterior wall of the inguinal canal without the use of synthetic mesh [8]. This method requires advanced surgical expertise but has demonstrated very low recurrence rates in specialized centers, such as the Shouldice Hospital [8]. In contrast, the Lichtenstein technique, introduced in the 1980s, relies on the placement of a synthetic polypropylene mesh to reinforce the inguinal canal, aiming to reduce tension and facilitate a technically simpler and reproducible repair [7, 9]. Consequently, Lichtenstein repair has become the most popular open approach globally [1, 2, 9].

Mesh-based repair has become the standard approach for most primary inguinal hernias due to its lower recurrence rates and technical reproducibility [1, 2, 7]. Current international guidelines from the HerniaSurge Group strongly recommend mesh-based techniques, such as the Lichtenstein or endoscopic repairs, as the primary approach for adult patients with inguinal hernias [2, 7]. However, the guidelines also state that tissue-based repairs, specifically the Shouldice technique, remain a viable option in the tailored approach for selected patients, particularly when the patient explicitly requests the avoidance of synthetic mesh, in contaminated fields, or when performed by surgeons with specific expertise in these techniques [2, 7].

Despite the widespread use of mesh repairs, concerns have been raised regarding mesh-related complications, including foreign body reaction, chronic inflammation, and nerve entrapment, which may contribute to a substantial incidence of chronic postoperative pain [4, 10, 11]. Chronic pain, defined as pain persisting beyond three months after surgery, affects approximately 10–30% of patients undergoing inguinal hernia repair and can severely impact daily activities, psychological well-being, and overall quality of life [3, 5].

Although previous studies have reported outcomes for both techniques, direct and robust comparisons focusing specifically on chronic postoperative pain are limited [4, 6]. Existing meta-analyses often pool heterogeneous surgical methods or lack recent data [4, 7]. Therefore, an updated and focused systematic review and meta-analysis comparing the Shouldice and Lichtenstein techniques with emphasis on chronic pain prevalence is warranted [3, 6].

The present study aims to provide an updated, comprehensive systematic review and meta-analysis comparing the Shouldice and Lichtenstein techniques, with a specific focus on the prevalence of chronic postoperative pain, while also evaluating hernia recurrence, operative time, and early postoperative complications. By elucidating these clinical and functional differences, our goal is to inform a principled tailored approach that prioritizes long-term quality of life and surgical efficacy in hernia repair [3, 6].

## Methods

This systematic review and meta-analysis were performed in accordance with the Cochrane Collaboration and the Preferred Reporting Items for Systematic Reviews and Meta-Analysis (PRISMA) statement guidelines [11]. The protocol for this review was registered in the International Prospective Register of Systematic Reviews (PROSPERO, ID: CRD420251058801).

### Search strategy and study selection

A comprehensive systematic literature search was conducted in the Medline (via PubMed), Embase, and Cochrane Library databases to identify relevant studies. The search strategy combined Medical Subject Headings (MeSH) and free-text terms related to “Shouldice,” “Lichtenstein,” “inguinal hernia,” “hernia repair,” and “chronic pain”. No language restrictions were applied; full details of the search strategy are available in Supplementary Material S1.

### Eligibility criteria

Studies were included if they met the following criteria: (1) Adult patients undergoing open inguinal hernia repair; (2) Direct comparison between Shouldice (non-mesh) and Lichtenstein (mesh) techniques; (3) Reporting prevalence or incidence of chronic postoperative pain, defined as pain persisting for more than 3 months post-surgery; (4) Randomized controlled trials (RCTs), prospective or retrospective cohort studies, and cross-sectional studies.

Exclusion criteria comprised: studies without direct comparison of the two techniques, pediatric populations, case reports, reviews, and those lacking adequate pain outcome data.

### Data extraction and quality assessment

The screening of titles and abstracts, full-text review for eligibility, data extraction, and quality assessment were all performed independently by two investigators. A standardized form was used for data extraction. Any disagreements during these stages were resolved through discussion and consensus, or by consultation with a third independent reviewer. Data extracted included: study design, sample size, patient demographics (age, sex), follow-up duration, mesh type, and the predefined clinical outcomes (chronic pain prevalence, recurrence rate, operative time, and other postoperative complications). The specific definitions, assessment tools, and time points used for chronic pain evaluation in each study were also systematically recorded.

Risk of bias was assessed using the Cochrane Risk of Bias 2.0 tool for RCTs and the Newcastle-Ottawa Scale (NOS) for observational studies [12, 13]. Domains assessed included selection bias, performance bias, detection bias, attrition bias, and reporting bias.

### Statistical analysis

Meta-analyses were conducted using Review Manager (RevMan) version 5.4.1. The primary outcome measure was the pooled relative risk (RR) with 95% confidence intervals (CI) comparing chronic pain prevalence between Shouldice and Lichtenstein groups. Heterogeneity was evaluated using the I² statistic; values of 25%, 50%, and 75% were considered low, moderate, and high heterogeneity, respectively. A fixed-effects model was applied when heterogeneity was I² ≤ 5%, and a random-effects model was considered otherwise.

Subgroup analyses were performed according to mean follow-up duration (< 3 years vs. ≥3 years) and study design (RCT vs. observational). Sensitivity analyses were conducted by sequentially excluding individual studies to assess the robustness of the results, and by removing studies with a high risk of bias.

## Results

### Study selection and characteristics

**Fig. 1:**
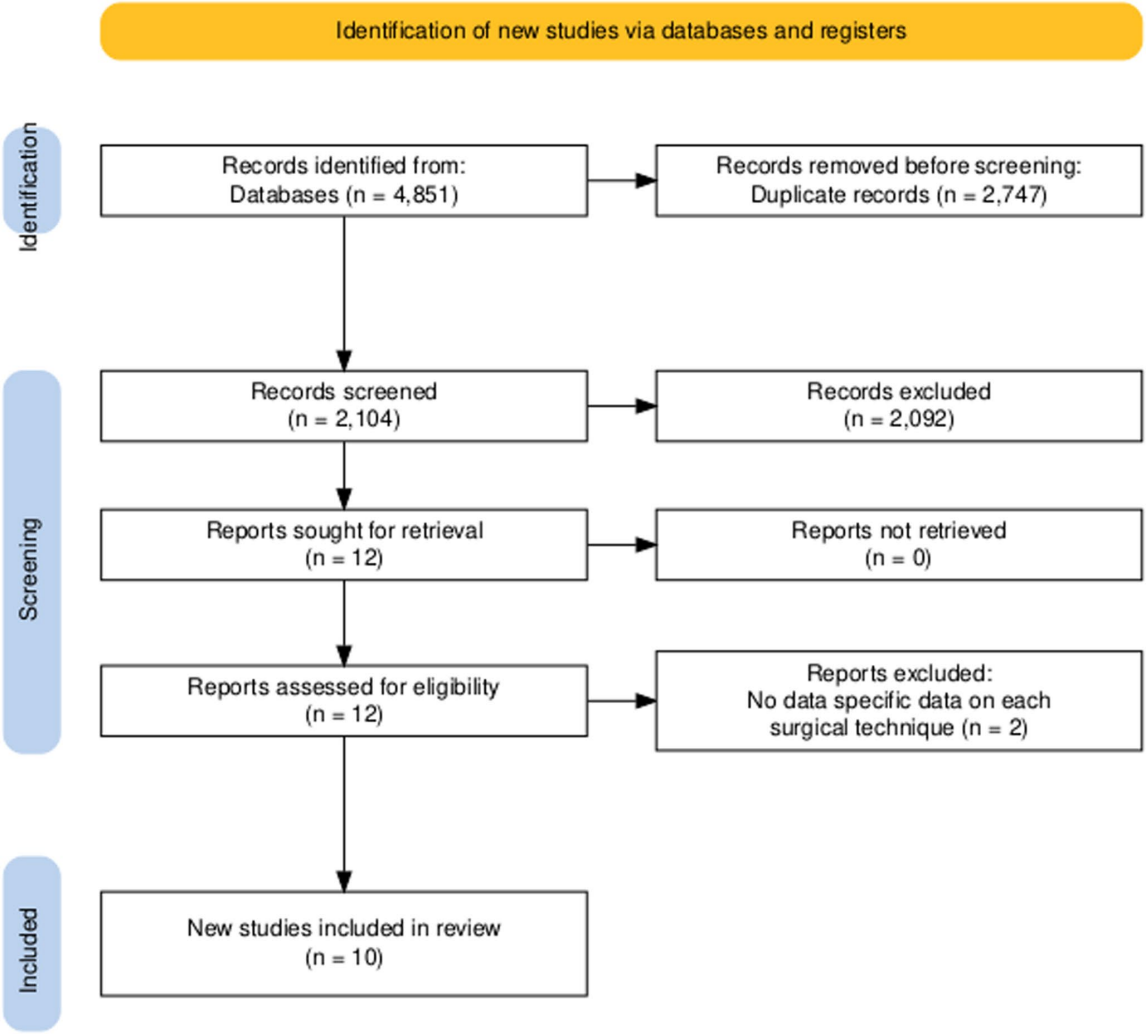
PRISMA flow diagram of study selection process

The systematic search identified 10 studies meeting the eligibility criteria, including seven randomized controlled trials and three observational studies, encompassing a total of 4,122 patients evaluated for the primary outcome. The search and selection process is summarized in the PRISMA flow diagram (Fig. 1). Among these patients, 2,466 (59.8%) underwent Lichtenstein repair and 1,656 (40.2%) underwent Shouldice repair. Study characteristics varied geographically and temporally, with follow-up durations ranging from 1 to 9 years. Baseline demographic characteristics, such as age and sex distribution, were comparable between surgical groups across the trials. Detailed baseline characteristics of the included studies are summarized in Table 1.

**Table 1:** Baseline characteristics of included studies

Study	Study Design	Total *N*	Shouldice (*n*)	Lichtenstein (*n*)	Follow-up (years)	Median Age (years)	Male (%)
Ahmadinejad (2024) [14]	Single-center RCT	401	183	218	3.0	S: 42.8 / L: 45.6	S: 94.0 / L: 92.7
Bay-Nielsen (2004) [15]	Population-based study	1880	630	1250	S: 2.6 / L: 2.1	< 55 (NR)	100
Miedema (2004) [16]	Prospective RCT	57	28	29	6.0–9.0	S: 62.0 / L: 63.0	100
Karakose (2016) [17]	Retrospective cohort	90	42	48	1.8	S: 29.0 / L: 46.0	S: 76.0 / L: 73.0
Königer (2004) [18]	Prospective RCT	150	74	76	4.3	S: 56.0 / L: 53.0	100
Wamalwa (2015) [19]	Single-center RCT	45	22	23	2.0	S: 63.5 / L: 60.0	100
McGillicuddy (1998) [6]	Prospective RCT	717	337	380	5.0	NR	100
Nordin (2002) [20]	Multicenter RCT	297	148	149	1.0	NR	100
Pokorny (2008) [21]	Multicenter RCT	143	74	69	3.0	S: 47.0 / L: 52.0	S: 89.0 / L: 93.0
Reinpold (2011) [22]	Prospective cohort	377	124	253	5.0	NR	88.6

Abbreviations: *RCT* randomized controlled trial, *S* Shouldice, *L* Lichtenstein, *NR* not reported

*Total N in* Table 1*refers to the initial enrolled/randomized populations. The final meta-analysis included 4*,*122 patients due to loss to follow-up in some trials*

**Table 2:** Absolute rates of clinical outcomes by study

Study	Chronic Pain *n*/*N* (%)		Recurrence *n*/*N* (%)		Other Complications *n*/*N* (%)	
	Shouldice	Lichtenstein	Shouldice	Lichtenstein	Shouldice	Lichtenstein
Ahmadinejad (2024)	4/183 (2.2%)	10/218 (4.6%)	18/183 (9.8%)	7/218 (3.2%)	10/183 (5.5%)	10/218 (4.6%)
Bay-Nielsen (2004)	119/630 (18.9%)	316/1250 (25.3%)	43/630 (6.8%)	80/1250 (6.4%)	NR	NR
Karakose (2016)	0/42 (0.0%)	2/48 (4.2%)	0/42 (0.0%)	0/48 (0.0%)	1/42 (2.4%)	0/48 (0.0%)
Königer (2004)	28/74 (37.8%)	25/76 (32.9%)	NR / 93	NR / 93	15/74 (20.3%)	13/76 (17.1%)
McGillicuddy (1998)	1/337 (0.3%)	4/371 (1.1%)	7/337 (2.1%)	2/371 (0.5%)	12/337 (3.6%)	6/371 (1.6%)
Miedema (2004)	2/28 (7.1%)	11/29 (37.9%)	2/41 (4.9%)	3/39 (7.7%)	NR	NR
Nordin (2002)	9/148 (6.1%)	8/149 (5.4%)	7/148 (4.7%)	1/149 (0.7%)	NR	NR
Pokorny (2008)	4/64 (6.3%)	4/66 (6.1%)	3/64 (4.7%)	0/65 (0.0%)	NR	NR
Reinpold (2011)	8/124 (6.5%)	44/233 (18.9%)	7/288 (2.4%)	14/493 (2.8%)	NR	NR
Wamalwa (2015)	0/22 (0.0%)	1/23 (4.3%)	0/22 (0.0%)	0/23 (0.0%)	0/22 (0.0%)	1/23 (4.3%)

**Table 3:** Definitions and assessment tools for chronic postoperative pain across included studies

Study	Assessment Tool	Time Point for Evaluation	Definition of Chronic Pain
Ahmadinejad (2024)	Visual Analogue Scale (VAS)	3 years	Pain persisting > 3 months postoperatively
Bay-Nielsen (2004)	Postal Questionnaire	2.1 to 2.6 years	Pain in the groin region restricting daily activities within the previous month
Karakose (2016)	Clinical evaluation / Chart review	21 months	Persistent pain reported during clinical follow-up
Königer (2004)	Visual Analogue Scale (VAS)	52 months	Pain lasting > 3 months, measured on a 100-point scale
McGillicuddy (1998)	Clinical examination / Interview	5 years	Discomfort or neuralgia persisting long-term
Miedema (2004)	Clinical examination / Telephone contact	7.1 to 7.3 years (mean)	Groin pain evaluated at physical or physician examination
Nordin (2002)	Physical examination / Questionnaire	1 and 3 years	Pain present at follow-up (assessed by an independent blinded examiner at 3 years)
Pokorny (2008)	Clinical examination / Visual Analogue Scale (VAS)	1, 2, and 3 years	Pain score > 0 reported at specific follow-up intervals
Reinpold (2011)	Visual Analogue Scale (VAS)	6 months and 5 years	Pain assessed systematically using VAS, compared to preoperative baseline
Wamalwa (2015)	Clinical assessment	2 years	Postoperative pain persisting after 3 months

### Prevalence of chronic postoperative pain

**Fig. 2:**
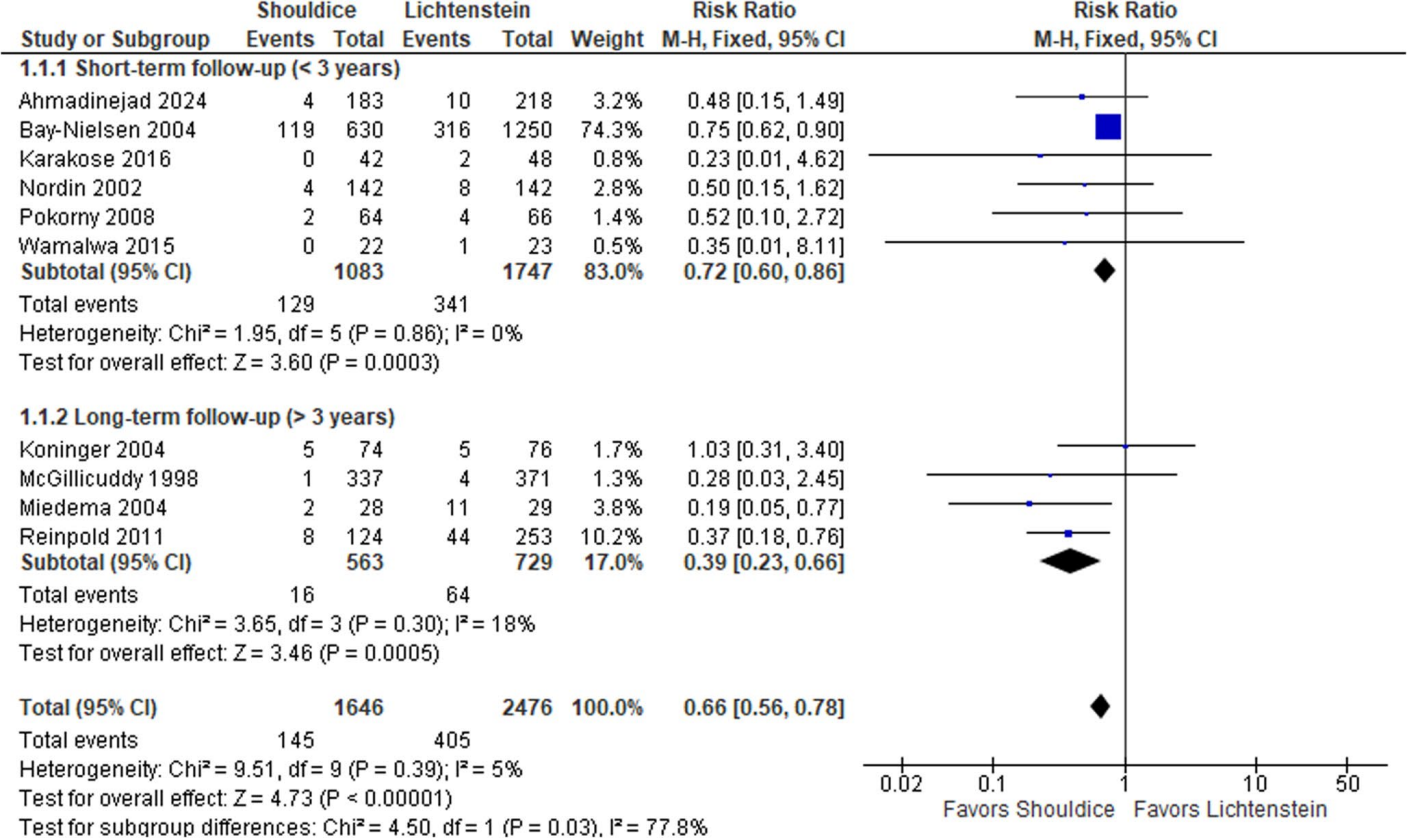
Forest plot comparing chronic postoperative pain prevalence between Shouldice and Lichtenstein repairs, stratified by mean follow-up duration

All included studies reported chronic pain (Table 2). While chronic pain is traditionally defined as pain persisting beyond three months postoperatively, the specific clinical definitions, assessment tools, and follow-up time points varied across the included studies, as systematically detailed in Table 3. The overall pooled analysis demonstrated that patients undergoing Shouldice repair had a significantly lower risk of chronic postoperative pain compared to those undergoing Lichtenstein repair (RR 0.66; 95% CI 0.56–0.78; *p* < 0.00001) (Fig. 2). The heterogeneity across studies was low (I² = 5%), indicating consistent findings.

### Subgroup analyses

**Fig. 3:**
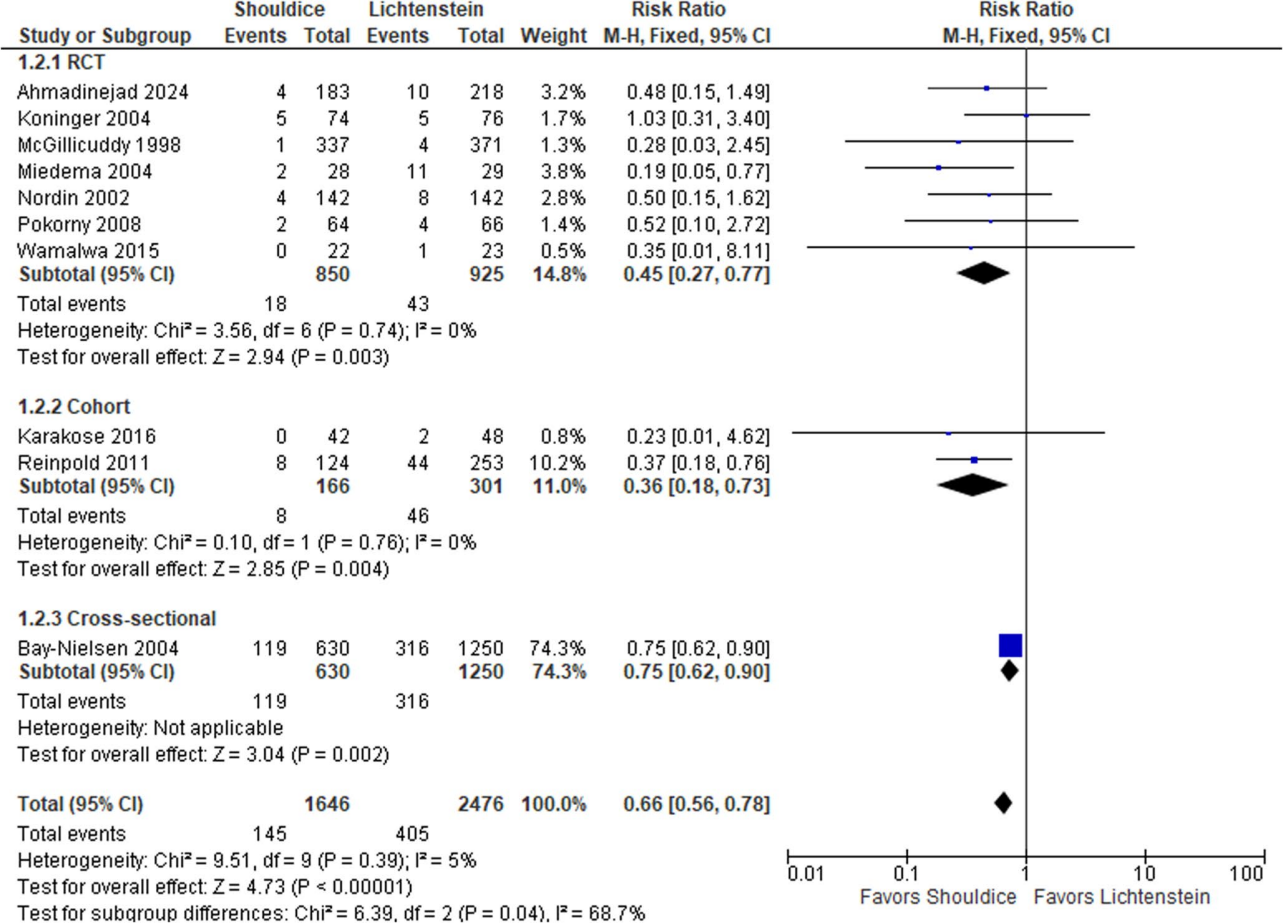
Forest plot comparing chronic postoperative pain prevalence between Shouldice and Lichtenstein repairs, stratified by study design

Subgroup analyses stratified by mean follow-up duration showed that the benefit of Shouldice repair in reducing chronic pain was consistent in studies with follow-up under 3 years (RR 0.72; 95% CI 0.60–0.86; *p* = 0.0003; I² = 0%) and those with follow-up of 3 years or more (RR 0.39; 95% CI 0.23–0.66; *p* = 0.0005; I² = 18%), suggesting durable long-term pain reduction (Fig. 3).

Further, stratification by study design revealed statistically significant lower chronic pain rates with Shouldice repair in both RCTs (RR 0.68; 95% CI 0.56–0.82; *p* < 0.0001; I² = 0%) and observational studies (RR 0.61; 95% CI 0.46–0.81; *p* = 0.0005; I² = 0%), reinforcing the robustness and generalizability of the findings.

### Recurrence Rate

**Fig. 4:**
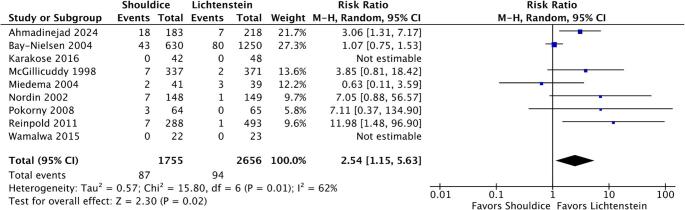
Forest plot comparing the recurrence rate between Shouldice and Lichtenstein repairs

Hernia recurrence rate was evaluated in nine studies, encompassing a total of 4,411 patients. The recurrence rate in the group undergoing the Shouldice technique ranged from 0% to 9.8% across trials, whereas in the Lichtenstein technique group, the variation was 0% to 7.7% (Table 2). The meta-analysis demonstrated that Shouldice repair is associated with a significantly higher risk of long-term recurrence compared to Lichtenstein repair (RR 2.54; 95% CI 1.15–5.63; *p* = 0.02; I² = 62%) (Fig. 4). The number of patients varied across outcomes due to differences in reporting and follow-up availability among studies.

### Other complications

**Fig. 5:**
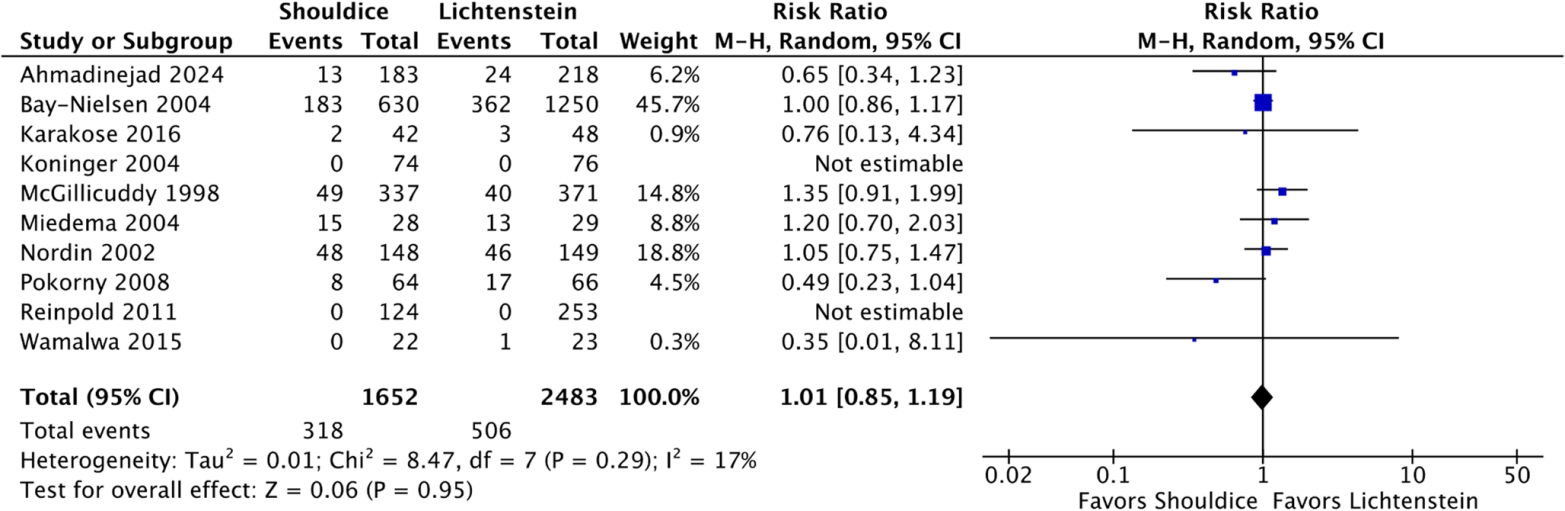
Forest plot comparing the rate of early postoperative complications between Shouldice and Lichtenstein repairs

The incidence of early postoperative complications was reported in ten studies (*n* = 4,135). The meta-analysis revealed no statistically significant difference in the overall rate of local adverse events between the two techniques (RR 1.01; 95% CI 0.85–1.19; *p* = 0.95; I² = 17%) (Fig. 5). Morbidity rates ranged from 0% to 10% in the Shouldice group and from 0% to 8% in the Lichtenstein group (Table 2). The complication profile predominantly consisted of superficial surgical site infection, hematoma or seroma formation, and acute urinary retention. It is noteworthy that superficial infection episodes were managed conservatively in all reports, with no need for deep surgical exploration or polypropylene mesh removal in the Lichtenstein arm. Furthermore, the occurrence of fluid collections and urinary retention was evenly distributed between both surgical approaches.

### Operative time

**Fig. 6:**
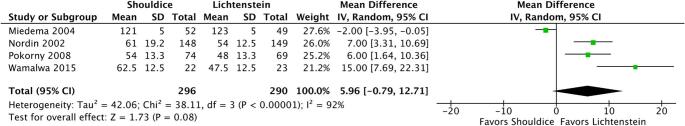
Forest plot comparing the operative time between Shouldice and Lichtenstein repairs

Four studies provided continuous data regarding surgical procedure duration, totaling 586 patients. The analysis demonstrated no statistically significant difference in mean operative time between the Shouldice technique and Lichtenstein repair (Mean Difference 5.96 min; 95% CI -0.79 to 12.71; *p* = 0.08; I² = 92%) (Fig. 6). The high heterogeneity likely reflects substantial variability in operative expertise and study settings.

### Risk of bias

**Fig. 7:**
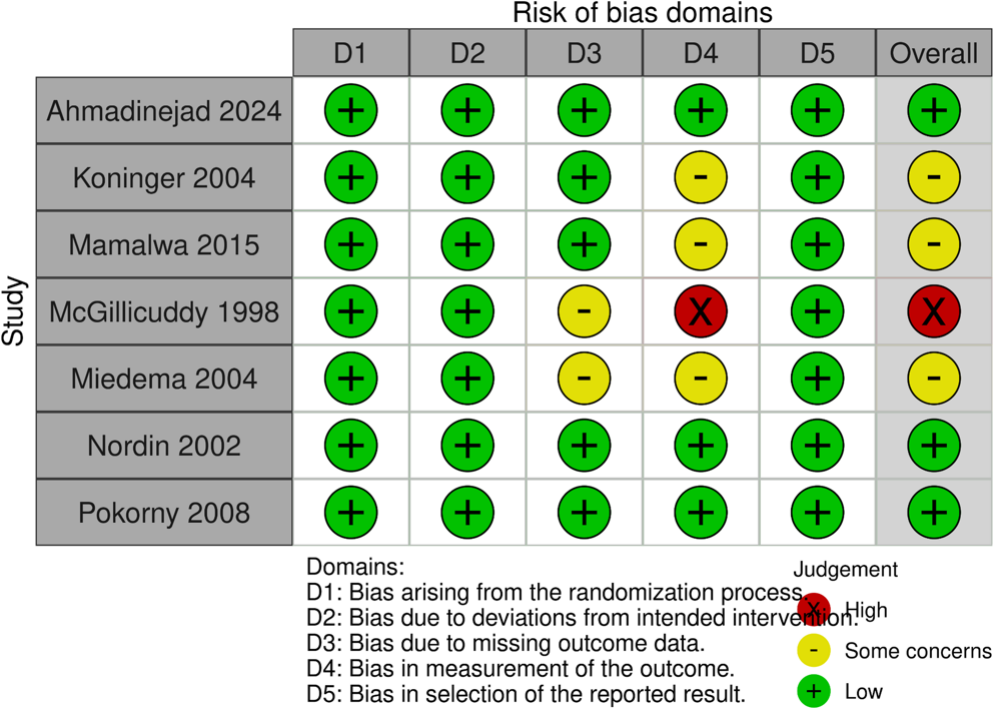
Risk of bias summary for the included randomized controlled trials evaluated using the Cochrane Risk of Bias 2 (RoB 2) tool

The methodological quality of the seven randomized controlled trials was assessed using the Cochrane Risk of Bias 2 (RoB 2) tool (Fig. 7). Two trials presented a low risk of bias, three raised ‘some concerns’ (primarily due to omissions in the description of the randomization process), and two were classified as having a high risk of bias, mainly because of critical flaws in randomization and deviations from intended interventions.

**Fig. 8:**
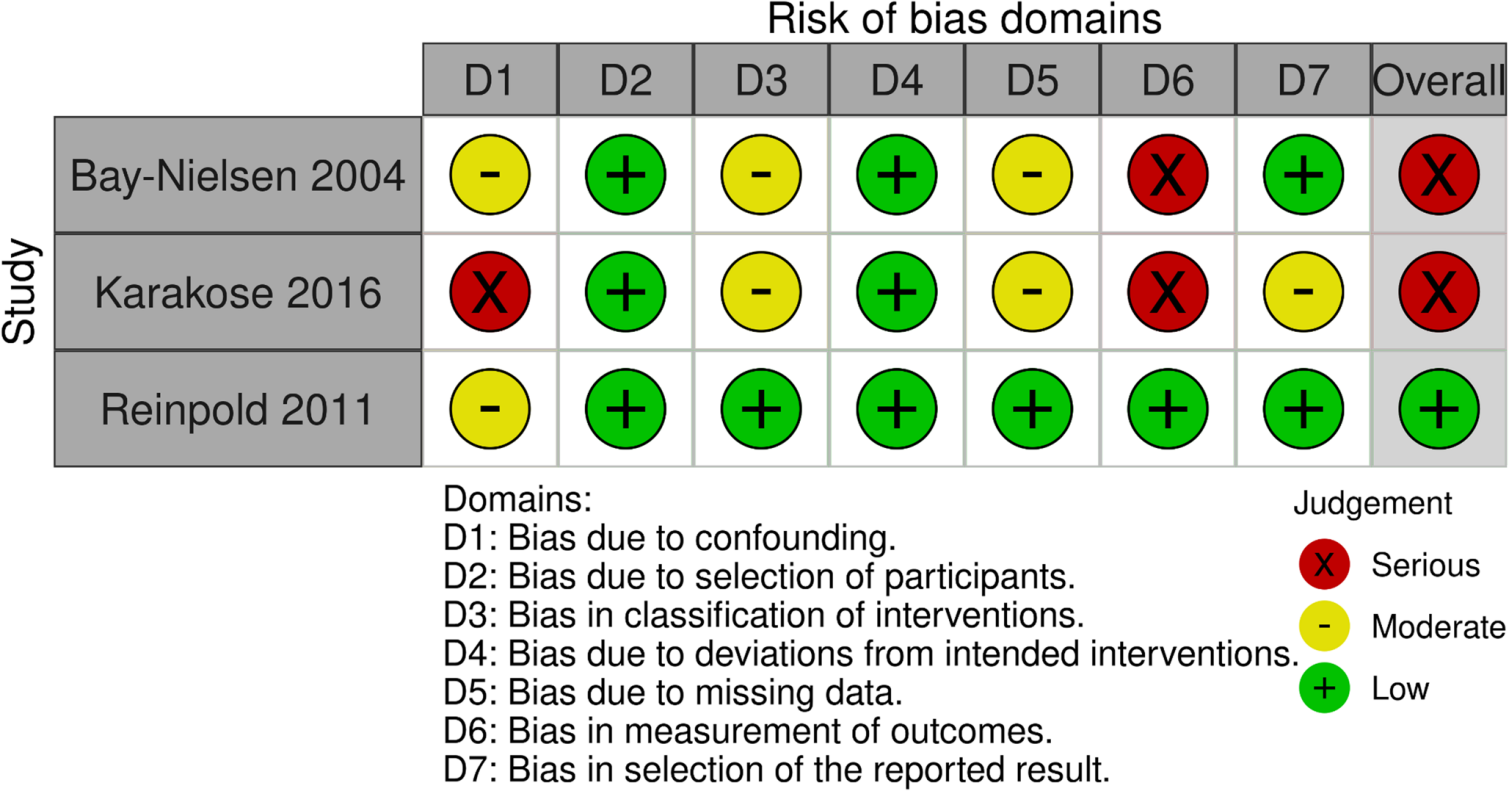
Risk of bias assessment for the included observational studies evaluated using the Newcastle-Ottawa Scale (NOS)

The three observational studies were evaluated using the Newcastle-Ottawa Scale (NOS), scoring between 5 and 8 points (out of a maximum of 9), which indicates moderate to high methodological quality (Fig. 8). The main limitations identified in these studies were concentrated in the selection and comparability domains. Funnel plot assessment was not performed because the number of studies was considered borderline for reliable interpretation.

### Sensitivity analyses

To ascertain the robustness of the findings, a sensitivity analysis was performed using the leave-one-out method. The sequential omission of individual trials did not alter the direction or statistical significance of the pooled effect for any of the evaluated outcomes. Similarly, the pre-specified exclusion of studies classified as having a high risk of bias maintained the consistency of the overall estimates, thereby reinforcing the validity of the results presented in this meta-analysis.

## Discussion

This systematic review and meta-analysis including 10 studies with 4,122 patients suggests that the Shouldice technique for open inguinal hernia repair is associated with a significantly lower risk of chronic postoperative pain compared to Lichtenstein mesh repair. The low heterogeneity observed (I² = 5%) suggests that this pain reduction is consistent across diverse populations.

To further contextualize the clinical significance of these findings, the absolute risk reduction for chronic postoperative pain was calculated at 6.5% in favor of the Shouldice technique. This translates to a Number Needed to Treat (NNT) of 15. In clinical terms, for every 15 patients treated with the Shouldice repair instead of the Lichtenstein technique, one additional case of long-term debilitating chronic pain may be prevented.

Chronic postoperative pain is a major long-term complication of inguinal hernia repair, severely impairing daily functioning and quality of life [15, 23, 24]. The present findings suggest that the tissue-based Shouldice repair, which avoids the implantation of synthetic mesh, may lessen the inflammatory and neuropathic mechanisms that contribute to persistent pain [22, 25, 26]. The meticulous, tension-free multilayered suture technique may also reduce nerve irritation and mesh-related foreign body reactions, thereby providing a plausible mechanistic explanation for the observed benefit.

While the Shouldice technique significantly mitigates chronic pain, our analysis also found a higher risk of long-term recurrence compared to the Lichtenstein repair. Notably, the recurrence outcome exhibited substantial heterogeneity (I² = 62%). This variability warrants a nuanced interpretation and is likely driven by factors such as patient selection, institutional volume, and, most importantly, surgeon experience. This is particularly relevant because the Shouldice repair is a technically demanding anatomical reconstruction with a steep learning curve. Although the Shouldice technique is highly standardized in the modern surgical literature, replicating the excellent structural outcomes reported by specialized high-volume centers likely requires dedicated training.

This reliance on surgical expertise, combined with differences in study settings (e.g., teaching hospitals versus high-volume specialized centers) and patient complexity, also explains the very high heterogeneity observed in mean operative time (I² = 92%). While our pooled data revealed no significant overall difference in operative duration, individual operative times likely varied according to surgeons’ familiarity with the non-mesh technique.

These findings support an individualized approach when selecting the surgical technique. Young, active patients with a low body mass index and small indirect hernias may be appropriate candidates for a non-mesh repair when performed by experienced surgeons, whereas older patients or those in whom a standardized, widely reproducible technique is preferred may be better served by a mesh-based approach [27–29]. Furthermore, proficient mastery of tissue repairs remains an important component of the general surgeon’s armamentarium in emergency settings. In cases involving bowel resection or gross contamination, where synthetic mesh placement carries a high risk of surgical site infection, the ability to perform rigorous anatomical reconstructions such as the Shouldice repair may be particularly valuable [30–33].

Limitations of the present review include the clinical heterogeneity in pain assessment methods, as previously detailed in Table 3. Blinding in surgical trials remains challenging, introducing risks of performance and detection bias. Moreover, the strict requirement for specific surgical expertise to achieve optimal results with the Shouldice technique limits the broad generalizability of our findings to non-specialized surgical practices. Finally, although the limited number of included studies (*n* = 10) precluded formal assessment of publication bias with funnel plot-based methods, publication bias cannot be entirely ruled out, as smaller studies with null results may have been less likely to be published.

## Conclusion

In conclusion, this meta-analysis demonstrates a clear clinical trade-off. Although the widely practiced Lichtenstein repair minimizes recurrence, the Shouldice technique yields significantly lower rates of chronic postoperative pain. Shouldice may be particularly suitable for selected patients, especially young, active individuals with low BMI and small indirect hernias, when performed by surgeons experienced in tissue-based repair. Furthermore, structured training in non-mesh techniques must be encouraged in surgical curricula to maintain a tailored, patient-centered standard. Balancing the impact of chronic pain against recurrence risk requires both shared decision-making and technical expertise in anatomical reconstructions.

## Supplementary Information

Below is the link to the electronic supplementary material.

Supplementary Material 1 - Detailed Search Strategy

## Data Availability

The datasets generated during and/or analyzed during the current study are available from the corresponding author on reasonable request.
