# Supplementary material for: Chronic pain after Shouldice versus Lichtenstein inguinal hernia repair: a systematic review and meta-analysis
Source: Hernia. 2026 May 12;30(1):213. doi: 10.1007/s10029-026-03702-x (PMC13167819; doi:10.1007/s10029-026-03702-x)
Supplement: Supplementary file 1 — Supplementary Material 1 - Detailed Search Strategy [file 10029_2026_3702_MOESM1_ESM.docx]

## Supplementary Material S1: Detailed Search Strategy

**General Overview**

The literature search was conducted across three primary electronic databases: Medline (via PubMed), Embase, and the Cochrane Central Register of Controlled Trials (CENTRAL). The search strategy was developed using a combination of Medical Subject Headings (MeSH), Emtree terms, and free-text keywords related to the surgical interventions and the outcome of chronic pain. No date or language restrictions were applied.

### 1. Medline (via PubMed)

- **#1 (Intervention):** ("mesh repair"[Title/Abstract] OR "non-mesh repair"[Title/Abstract] OR "hernioplasty"[Title/Abstract] OR "herniorraphy"[Title/Abstract] OR "Lichtenstein"[Title/Abstract] OR "Shouldice"[Title/Abstract] OR "hernia repair"[Title/Abstract] OR "Herniorrhaphy"[Mesh] OR "Hernia Repair"[Mesh])
- **#2 (Outcome):** ("chronic pain"[Title/Abstract] OR "CPHP"[Title/Abstract] OR "neuropathic"[Title/Abstract] OR "neuralgia"[Title/Abstract] OR "post-herniorrhaphy pain"[Title/Abstract] OR "persistent pain"[Title/Abstract] OR "chronic post-surgical pain"[Title/Abstract] OR "chronic postoperative pain"[Title/Abstract] OR "inguinodinia"[Title/Abstract] OR "Chronic Pain"[Mesh] OR "Neuralgia"[Mesh])
- **#3 (Final Search):** #1 AND #2

### 2. Embase

- **#1 (Intervention):** ('mesh repair':ti,ab,kw OR 'non-mesh repair':ti,ab,kw OR 'hernioplasty':ti,ab,kw OR 'herniorraphy':ti,ab,kw OR 'Lichtenstein':ti,ab,kw OR 'Shouldice':ti,ab,kw OR 'hernia repair':ti,ab,kw OR 'herniorrhaphy'/exp)
- **#2 (Outcome):** ('chronic pain':ti,ab,kw OR 'CPHP':ti,ab,kw OR 'neuropathic':ti,ab,kw OR 'neuralgia':ti,ab,kw OR 'post-herniorrhaphy pain':ti,ab,kw OR 'persistent pain':ti,ab,kw OR 'chronic post-surgical pain':ti,ab,kw OR 'chronic postoperative pain':ti,ab,kw OR 'inguinodinia':ti,ab,kw OR 'chronic pain'/exp OR 'neuralgia'/exp)
- **#3 (Final Search):** #1 AND #2

### 3. Cochrane Library (CENTRAL)

- **#1 (Intervention):** ("mesh repair" OR "non-mesh repair" OR hernioplasty OR herniorraphy OR lichtenstein OR shouldice OR "hernia repair"):ti,ab,kw
- **#2 (Outcome):** ("chronic pain" OR "CPHP" OR "neuropathic" OR "neuralgia" OR "post-herniorrhaphy pain" OR "persistent pain" OR "chronic post-surgical pain" OR "chronic postoperative pain" OR inguinodinia):ti,ab,kw
- **#3 (Final Search):** #1 AND #2
